# Cognitive task analysis-based training in surgery: a meta-analysis

**DOI:** 10.1093/bjsopen/zrab122

**Published:** 2021-12-14

**Authors:** Thomas C Edwards, Alexander W Coombs, Bartosz Szyszka, Kartik Logishetty, Justin P Cobb

**Affiliations:** MSk Lab, Imperial College London, London, UK; MSk Lab, Imperial College London, London, UK; MSk Lab, Imperial College London, London, UK; MSk Lab, Imperial College London, London, UK; MSk Lab, Imperial College London, London, UK

## Abstract

**Background:**

Reduced hands-on operating experience has challenged the development of complex decision-making skills for modern surgical trainees. Cognitive task analysis- (CTA-)based training is a methodical solution to extract the intricate cognitive processes of experts and impart this information to novices. Its use has been successful in high-risk industries such as the military and aviation, though its application for learning surgery is more recent. This systematic review aims to synthesize the evidence evaluating the efficacy of CTA-based training to enable surgeons to acquire procedural skills and knowledge.

**Methods:**

The PRISMA guidelines were followed. Four databases, including MEDLINE, EMBASE, Web of Science and Cochrane CENTRAL, were searched from inception to February 2021. Randomized controlled trials and observational studies evaluating the training effect of CTA-based interventions on novices' procedural knowledge or technical performance were included. Meta-analyses were performed using a random-effects model.

**Results:**

The initial search yielded 2205 articles, with 12 meeting the full inclusion criteria. Seven studies used surgical trainees as study subjects, four used medical students and one study used a combination. Surgical trainees enrolled into CTA-based training groups had enhanced procedural knowledge (standardized mean difference (SMD) 1.36 (95 per cent c.i. 0.67 to 2.05), *P* < 0.001) and superior technical performance (SMD 2.06 (95 per cent c.i. 1.17 to 2.96), *P* < 0.001) in comparison with groups that used conventional training methods.

**Conclusion:**

CTA-based training is an effective way to learn the cognitive skills of a surgical procedure, making it a useful adjunct to current surgical training.

## Teaser

Cognitive task analysis- (CTA-)based training is an innovative solution to teaching complex surgeries outside of the operating room. This meta-analysis evaluates the effectiveness of CTA-based training to acquire procedural knowledge and technical skills compared with conventional training methods.

## Introduction

Mastery in surgical disciplines requires development of both technical proficiency and refined surgical judgement. Some authors suggest this process may take in excess of 10 000 hours of deliberate practice to achieve^1^. With restrictions on trainee working hours seen across the globe, supervised learning experiences in the operating room (OR) are diminishing. A recent study noted a 25 per cent reduction in first-year trainee operative cases following the introduction of a 16-hour duty maximum, when compared with the preceding 4 years^2^. These restrictions and a decline in trainee autonomy in the OR, have led to concerns regarding the readiness of final-year trainees for independent practice^3^. To ensure patient safety is not compromised, there is a clear need to develop innovative solutions to educate trainees more efficiently, achieving the required level of competence with less hands-on operating time.

In order to expedite the development of technical skills, several promising simulation technologies have been developed, with many demonstrating effective skills transfer to the OR^4^^,^^5^. Surgical judgment and decision-making skills are more difficult to learn outside the OR, and require many years of experience to develop and refine. Cognitive task analysis (CTA), a technique developed by the military, involves systematically extracting the cognitive processes behind each key step of a procedure using experts. The technique, as described by Militello and colleagues, involves direct observation and structured interviews where multiple experts are probed on the task sequence, key decision points and rationales, technical tips, potential pitfalls and their remedies^6^^,^^7^. Using this information, procedure-based CTA curricula can be developed and amalgamated into various multimedia platforms or utilized in more traditional training modalities, such as attending in-person courses. This approach has proven to be an efficacious method to gain competence rapidly and accelerate long learning curves^8^.

Over the last decade, CTA methodology and the technology with which this training is delivered have developed considerably. As an easily accessible, cost-effective learning modality, CTA-based training could become a valuable solution to help expedite trainees’ progress towards competence. This meta-analysis investigates whether CTA-based training is more effective than conventional training in delivering technical skills and procedural knowledge to novices. The hypothesis is that this innovative strategy will be more effective than conventional methods.

## Methods

### Data sources and search strategy

A systematic review was conducted in accordance with the PRISMA guidelines (*Fig. S1*)^9^. Four databases were searched including MEDLINE and EMBASE through OVID, Web of Science and The Cochrane Library (CENTRAL). Databases were searched from inception to 3 February 2021. Search terms for MEDLINE were: (Cognitive task analysis.mp. OR Cognitive training.mp. OR Cognitive adj2 simulation.mp. OR Cognitive skills.mp.) AND (Surg*.mp. OR Operat*.mp.). The terms were adapted for other databases (*Table S1*) and a search of the grey literature was also performed (*Table S2*). The reference lists of included articles were examined for additional eligible studies.

### Inclusion and exclusion criteria

Studies investigating the impact of any CTA-based training tool on educating novices in surgical procedures were included. CTA-based training tools were defined as training interventions where CTA methodology was used to design its content. A surgical procedure in this study was defined as any invasive procedure related to surgery that involves multiple discrete steps. All surgical specialties were included. The study population were novices in the procedure including surgical trainees or medical students. Studies were excluded if the training effect of the CTA intervention was not quantified, if training interventions were not developed using CTA methodology and if the articles were in languages other than English. The full inclusion and exclusion criteria are listed in *Table 1*.

**Table 1:** Inclusion and exclusion criteria

Category	Criteria	
	Inclusion	Exclusion
**Study design**	Primary original research Randomized controlled trials Non-randomized controlled trials Observational studies Cohort and case-control studies Cross-sectional studies	Systematic reviews Meta-analyses Case reports
**Population**	Surgical trainees Novices: Medical students Clinician not in specialist training pathways	Expert surgeons only (consultant/attending physician level) Publications with overlapping participant population (same participants but different studies)
**Intervention**	CTA-based training teaching any surgical procedure: CTA tools CTA-based curriculum CTA apps	Studies whereby the CTA intervention is not isolated for effect (e.g. uses CTA intervention with additional other methods of training not used in the control)
**Control intervention**	Non-CTA methods Conventional methods No control	No restriction criteria
**Outcomes**	Procedural knowledge or technical performance of surgical procedure	Studies that do not explore either procedural knowledge or technical performance of surgical procedure
**Publication type**	Fully peer-reviewed publications Grey literature: Conference papers Dissertations Unpublished trials on registered trial databases Undergoing trials	Publications reporting abstracts only Publications reporting non-primary data
**Date of search**	No restriction criteria	No restriction criteria
**Geographical location**	No restriction criteria	No restriction criteria
**Publication language**	English	All languages except English

CTA, cognitive task analysis.

### Study selection and data extraction

Following the removal of duplicate studies, two investigators screened the titles and abstracts independently for relevance. Subsequently, the same investigators examined the full texts of the identified articles using the inclusion criteria in *Table 1*. Data were extracted according to a pre-agreed protocol which included study design, surgical procedure, CTA method, CTA intervention(s), control(s), assessment method, outcome measurements and the summary of results. Conflicts in study selection and data extraction were resolved through discussion with a third investigator.

### Assessment of bias and evidence quality

Risk of bias was assessed by two investigators independently, using Cochrane’s risk of bias for randomized controlled trials tool (RoB 2) and Cochrane’s risk of bias in non-randomized studies of interventions tool (ROBINS-I)^10^^,^^11^. The methodological quality of each study was also assessed independently by the same investigators using the Medical Education Research Study Quality Instrument (MERSQI)^12^. The MERSQI consists of 10 items in six domains, with scores ranging from a minimum of five to a maximum of 18. In this study scores of 5.0–9.5 were considered low quality, 10.0–13.5 were moderate quality and 14.0–18.0 were high quality. All conflicts were resolved through discussion with a third investigator.

### Data analysis

Where three or more randomized controlled trials reported the same outcome, and data were presented in the correct format, a meta-analysis was performed. Observational studies were excluded from the meta-analysis. The outcomes of interest were procedural knowledge (scores from multiple-choice question (MCQ) tests and talk-aloud protocols) and technical performance (global rating scores). A random effects model was employed where data were considered to have moderate heterogeneity (*I^2^* > 30 per cent)^13^. Where heterogeneity was considerable (*I^2^* > 75 per cent)^13^, a sensitivity analysis was performed, examining the impact of removing studies with high risk of bias or where information about the CTA methodology employed was omitted, and grouping studies by population. Where similar outcomes were measured using different scales, an inverse variance analysis was performed for continuous variables reporting the standardized mean difference (SMD) with 95 per cent confidence intervals. *P* < 0.050 was considered significant. Statistics were performed using Reviewer Manager (RevMan), version 5.4 (The Nordic Cochrane Centre, The Cochrane Collaboration, Copenhagen, Denmark).

## Results

### Search results

**Fig. 1:**
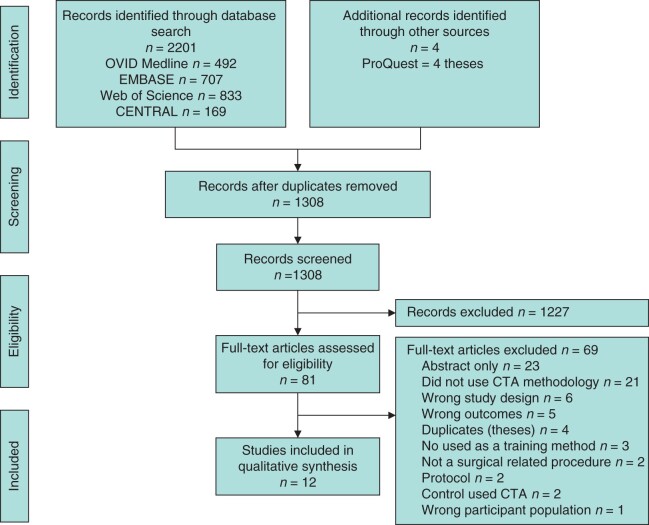
PRISMA flow diagram

The initial search result yielded 2205 articles. Full texts of 81 relevant studies were screened, of which 12 met the inclusion criteria^14–25^. There were no additional studies found from examining the reference lists of included studies. The grey literature search identified four theses relevant to the review, but all were excluded as duplicates due to being recognized as identical papers to published literature found in full-text screening^26–29^. The PRISMA flow chart summarizing this process is displayed in *Fig. 1*.

### Study characteristics

Of the 12 included articles, 10 were randomized controlled trials and two were observational studies (*Table S3*). In total, 327 (mean 27) participants were enrolled into the studies, including 162 surgical trainees and 139 medical students. Only one study (26 participants) included both surgeons and students. Five studies evaluated CTA-based training in a trauma and orthopaedics procedure^15^^,^^16^^,^^19^^,^^22^^,^^25^, three in otolaryngology^14^^,^^17^^,^^23^, two in general surgery^18^^,^^21^, one in plastic surgery^20^ and the remaining study was related to a generic surgical skill^24^.

### Cognitive task analysis-based training techniques

The CTA methodology used to elicit the expert knowledge was reported in seven of the articles^15–17^^,^^19^^,^^20^^,^^23^^,^^24^. All seven performed interviews with more than one expert to identify the procedural steps and cognitive decision points of the surgical procedure. Three studies opted for a modified Delphi approach to gain consensus^15^^,^^16^^,^^19^, whereas the other four studies either performed a series of documentational reviews similar to Delphi or used discussion methods to agree on a consensus final document^17^^,^^20^^,^^23^^,^^24^. CTA-based training interventions were delivered using courses (one study)^14^^,^^24^, curricula (one study)^17^^,^^23^, computer software (one study)^15^^,^^20^, web-based multimedia tools (two studies)^16^^,^^19^^,^^21^ and mobile phone applications (two studies)^18^^,^^22^^,^^25^.

### The impact of cognitive task analysis-based training on procedural knowledge

Five studies assessed the impact of CTA-based training on procedural knowledge without a control arm^16^^,^^20–22^^,^^25^. Procedural knowledge was most commonly assessed by an MCQ test or a talk-aloud protocol—a verbal examination where study participants guide examiners through their thought processes behind the performance of a procedure. All five studies reported a statistically significant improvement of procedural knowledge after training^16^^,^^20–22^^,^^25^.

Five studies assessed the procedural knowledge of novices using CTA-based training *versus* conventional training methods^16^^,^^17^^,^^19^^,^^23^^,^^24^. Four studies reported significantly superior procedural knowledge when compared with the control group. Campbell and colleagues reported equivalent performance of cricothyrotomy in participants who had received CTA-based training or a traditional curriculum^17^.

**Fig. 2:**
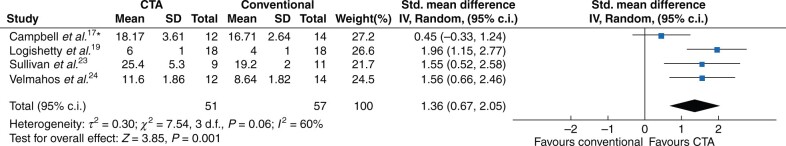
Forest plot of randomized controlled trials for procedural knowledge assessment scores comparing surgical trainees undergoing cognitive task analysis-based training versus conventional training SD, standard deviation; Std. Mean Difference, standardized mean difference; IV, inverse variance; CTA, cognitive task analysis. *Studied population was a combination of trainees and medical students.

Five studies were eligible for meta-analysis, one study was excluded because means and standard deviations were not reported. The remaining four studies had moderate heterogeneity (*I^2^* = 60 per cent) so a random effects model was used. The SMD between the groups for procedural knowledge scores was 1.36 (95 per cent c.i. 0.67 to 2.05), *P* < 0.001), significantly in favour of CTA-based training (*Fig. 2*).

### The impact of cognitive task analysis-based training on technical performance

Seven studies assessed the technical performance of novices using CTA-based training *versus* conventional training methods. Performance was assessed on simulators, synthetic models or real patients^14^^,^^15^^,^^17–19^^,^^23^^,^^24^. Five of these studies reported significantly superior technical performance in the CTA-based trained group^15^^,^^17^^,^^19^^,^^23^^,^^24^. The two remaining studies were those with only medical students as study participants^14^^,^^18^. A more detailed review of the results for the included studies are displayed in *Table S4*.

**Fig. 3:**
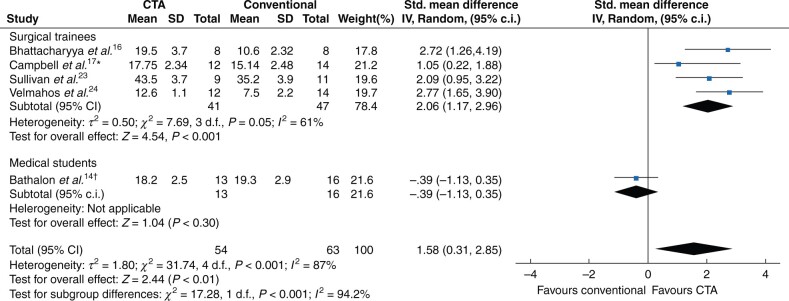
Forest plot of randomized controlled trials for technical performance measured using global rating scores comparing cognitive task analysis-based and conventional training, grouped by studied population (surgical trainees or medical students) SD, standard deviation; Std. mean difference, standardized mean difference; IV, inverse variance; CTA, cognitive task analysis. *Studied population was a combination of trainees and medical students. †Study did not report CTA methodology.

Two articles did not use global rating scores in their assessment of novices and were therefore excluded from the meta-analysis. The heterogeneity of the included studies was considerable (*I^2^* = 87 per cent), so a sensitivity analysis was performed and a random effects model was used. Two factors were considered: analysing medical students and surgical trainees separately, and removing studies where the CTA methodology used to derive the intervention was not documented. Sensitivity analysis suggested analysing studies by population reduced the heterogeneity and so the medical student study (Bathalon and colleagues) was removed and analysed separately^14^. The medical student study was also the only included study that did not state the CTA methodology used. The heterogeneity of the trainee studies was moderate (*I^2^* = 61 per cent). Subgroup analysis of the remaining four trainee studies favoured CTA-based training to improve technical performance (SMD 2.06 (95 per cent c.i. 1.17 to 2.96), *P* < 0.001). When the medical student study was included, the total effect was attenuated but was still in favour of CTA-based training (SMD 1.58 (95 per cent c.i. 0.31 to 2.85), *P* = 0.010) as demonstrated in *Fig. 3*.

### Risk of bias

**Fig. 4:**
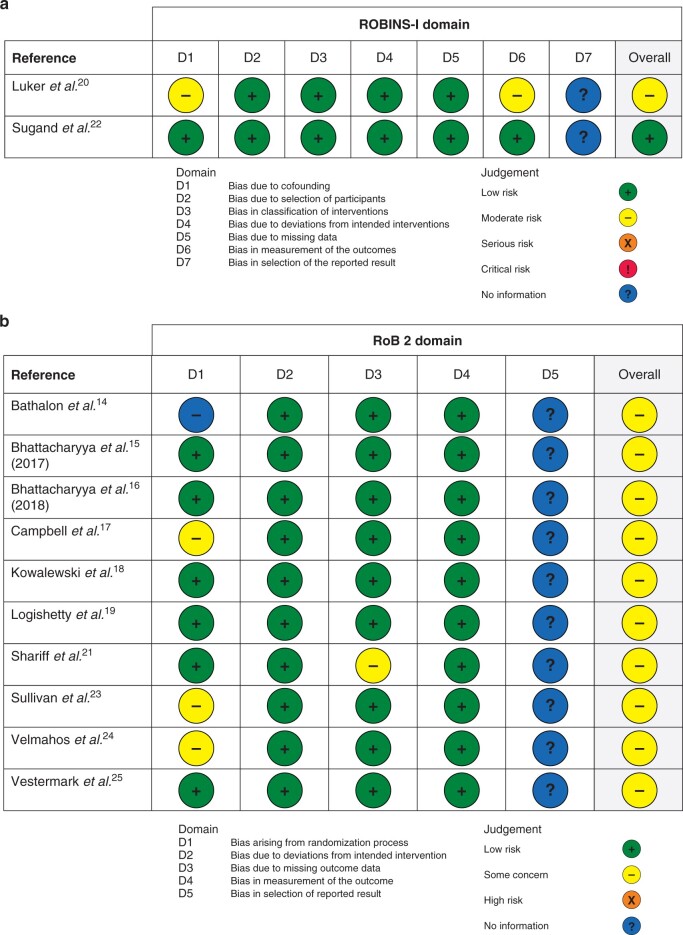
Cochrane risk of bias for included studies **a** Cochrane’s risk of bias in non-randomized studies of interventions tool (ROBINS-I). **b** Risk of bias for randomized controlled trials tool (RoB 2)

A visual depiction of the risk-of-bias assessment for each study is presented in *Fig. 4*. While most domains were ‘low risk’, the risk of bias overall for the included studies was found to be of ‘some concern’. This was due to the lack of availability of study protocols, and potential bias in the selection of the reported result domain.

### Medical Education Research Study Quality Instrument score

On average, the included articles were of a moderate quality (mean(s.d.) MERSQI score 13.6(1.35), range 11.0 to 15.5). Six studies were of high methodological quality^15^^,^^16^^,^^18^^,^^19^^,^^21^^,^^24^ with the remaining six of moderate quality^14^^,^^17^^,^^20^^,^^22^^,^^23^^,^^25^ (*Table S5*). No study, aside from Bhattacharyya and colleagues—who assessed the effect of CTA-based training on learning knee arthroscopy—achieved maximum points for the validity of evaluation instrument domain^15^. Velmahos *et al.* (CTA-based training for central venous catheterization) were the only authors to report outcomes related directly to patient care^24^.

## Discussion

The principal finding of this meta-analysis was that the innovative CTA-based training approach was significantly more effective than conventional training, in allowing surgical trainees to develop procedural knowledge and technical skills. Given the global reduction in hands-on operating during training and growing concerns regarding final year trainees’ readiness for independent practice, this new approach could be used as an adjunct to OR training, accelerating the development of competence^2^^,^^3^.

The large portion of high-quality randomized controlled trials, combined with the significant effect observed, further strengthens the interpretation of the results. Cohen’s definition suggests that SMDs of greater than 0.8 are considered large effect sizes^30^. The observed SMDs of 2.06 for technical performance and 1.36 for procedural knowledge therefore indicate a substantial benefit of CTA-based training over conventional methods. The clear distinction of procedural steps and its emphasis on cognitive decision points provides learners with a structured framework to process the knowledge of a difficult surgical procedure. Furthermore, it is suggested the inclusion of those automated principal steps is advantageous to learners in gaining a more detailed procedural understanding compared with conventional training material^31–33^. Surgical trainees may therefore use CTA-based training to assist their education during the ascent stage of the learning curve.

Previous studies have suggested the teaching of cognitive skills should occur prior to training in technical psychomotor skills^34^. The current study supports this recommendation, as does Fitts and Posner’s three-stage theory of motor-skill acquisition. In this model, the trainee first must comprehend the process of the procedure in the initial cognitive stage before any repeated practice of that skill can take place^35^. CTA-based training is thought to supplement this cognitive stage of the model by increasing the trainee’s baseline knowledge which provides them with a moderate degree of cognizance when performing the procedure for the first time. Additionally, it has been reported in a systematic review by Hull and colleagues that non-technical skills, such as cognitive decision-making, can enhance the performance of technical skills^36^.

An important consideration in any surgical education study is whether any beneficial training effect seen translates into improved patient outcomes. Whilst the majority of the studies were assessed in a simulated environment, Velmahos and co-workers did assess technical performance of trainees performing central venous catheterization using real-life patients^24^. The authors demonstrated superior technical performance and knowledge, with no complications in the intervention group, compared with the two complications found in the conventionally trained group, suggesting the possibility of improved patient safety using the CTA-based training. Whilst this study may have been underpowered to detect differences in complications, the link between superior technical performance and improved patient outcomes has been well established^37^. In a recent study, Stulberg and colleagues demonstrated surgeons who exhibited better technical performance had fewer complications, reoperations, deaths and less serious morbidity when performing a laparoscopic right hemicolectomy^38^. These findings have been supported by a multispecialty systematic review by Fecso, suggesting a strong link between surgeons’ technical performance and patient outcomes in 21 out of the 24 included studies^37^. Furthermore, effective decision-making was one of the main components recommended by Regenbogen and co-workers to avoid surgical technical error^39^. The lack of autonomy and independence during training may further hinder the development of these decision-making skills and subsequent progress to safe independent practice^3^. CTA-based training may therefore accelerate this development, which may in turn lead to improved patient outcomes. This is an interesting area for further investigation.

CTA-based training was highly efficacious in educating surgical trainees, but it did not appear to be as effective when applied to the medical student population. Bathalon and colleagues showed no significant differences in technical performance between CTA-based and conventionally trained medical students performing an open cricothyrotomy^14^. Similarly Kowalewski and colleagues demonstrated no difference in their study assessing medical students performing a laparoscopic cholecystectomy^18^. In contrast, the surgical trainee studies all demonstrated a significant benefit of the CTA-based training. This perhaps suggests some prior knowledge of the procedure and basic surgical skills may be necessary before more difficult procedural and cognitive decision learning is applied. The theory of constructivism proposed by Piaget may rationalize this finding^40^. Constructivist theory is the belief that the learning of new knowledge and skills is developed from a framework of pre-existing knowledge on the subject matter^41^. In context, this suggests medical students must first have some experience within surgery and the OR in order to attain those complex learning points as provided by the CTA-based training. Surgical trainees on the other hand, were able to build on to their pre-existing knowledge to advance their surgical performance rapidly using the CTA-based training. This finding questions the validity of using medical students to assess the effectiveness of these interventions. This issue has been raised by Cook, who indicated spectrum bias may be seen in studies where the assessed participant group used to validate the instrument will ultimately be different to the population for which it is intended^42^. Given the findings of this study, the use of medical students to assess technical performance in procedures that only postgraduates execute may limit the generalizability of the results and subsequent application to surgical trainees. Future research evaluating surgical education interventions should consider using an appropriate study population to avoid potentially misrepresenting the impact of their intervention.

Another consideration is the medium through which the CTA-based training was delivered. In studies published before 2014, courses and curricula were predominantly used. Subsequently, most of the CTA-based training was developed for use on an electronic device such as a computer or mobile phone application. This process of delivering training is favoured as electronic devices are easily accessible to all modern trainees and, unlike courses, can be revisited frequently for consolidation—a process considered essential to the transfer and retainment of procedural knowledge and skills in surgery^43^^,^^44^. Mobile phone applications deliver CTA-based training through interactive simulation of the procedure and use ‘serious gaming’ to quiz the user’s knowledge^18^. CTA-based training can also be amalgamated into a web-based multimedia tool. These tools are potentially more comprehensive, allowing more detail and visual material. The caveat is that they may not be as immediately accessible as a mobile phone application which may limit the number of repetitions and consolidation of the acquired knowledge. Several studies demonstrate web-based tools and serious gaming have a superior training effect when compared with conventional methods, further supporting their use in delivering CTA-based training to surgical trainees^45^^,^^46^.

There are some limitations to this study. First, despite there being 12 studies with ten randomized controlled trials meeting the inclusion criteria, only five were included in the meta-analysis. This combined with the overall heterogeneity of the data, procedures included and methods of assessment, may limit the interpretation of the results. Another important limitation of this study was the variety of platforms through which the CTA-based training and conventional training was delivered. For example, in the study by Bhattacharyya and colleagues, the control group used an operative technical manual, whereas the CTA-based intervention group used a web-based multimedia tool^16^. It is therefore difficult to determine if the positive training effect was exclusively due to the interactive multimedia or due to the CTA content. On the contrary, in a study by Shariff, no differences were found between groups who learnt using a CTA-based study day compared with a CTA-based multimedia tool, suggesting this may not be a significant confounder^21^.

Another limitation was the lack of pre-intervention baseline testing of procedural knowledge and technical performance in some of the included studies. Without this, even if baseline characteristics are equivalent between groups, it is still difficult to establish whether the positive effect seen after intervention could be a result of potential imbalances in baseline knowledge and ability.

Finally, the detailed reporting of CTA methodologies only featured in seven of the 12 articles^15–17^^,^^19^^,^^20^^,^^23^^,^^24^. Most notably, studies using surgical simulation app, Touch Surgery™ (Digital Surgery Limited, London, United Kingdom), did not report any detail on how the CTA content was derived and then implemented into the training module^18^^,^^22^^,^^25^. In order to understand what CTA methods may work best for designing CTA-based training, the methodology must be described completely in publications to allow robust comparison.

This study suggests CTA-based training is superior to conventional training in developing procedural knowledge and technical proficiency in surgical procedures. The substantial training effect demonstrated in a surgical-trainee population was attenuated when applying this training strategy to medical students, questioning the validity of using medical students in evaluating training interventions aimed at surgeons. Based on the presented evidence, training-programme leaders should strongly consider integration of CTA-based training into the current surgical curriculum.

## Supplementary Material

zrab122_Supplementary_Data
