# Supplementary material for: Cognitive task analysis-based training in surgery: a meta-analysis
Source: BJS Open. 2021 Dec 14;5(6):zrab122. doi: 10.1093/bjsopen/zrab122 (PMC8669793; doi:10.1093/bjsopen/zrab122)
Supplement: zrab122_Supplementary_Data [file zrab122_supplementary_data.zip › Supplementary_Tables_1_and_2.docx]

| **Supplementary Table 1.** Search strategy for databases | | | |
| --- | --- | --- | --- |
| Database | **#** | **Search terms** |  |
| **MEDLINE** | 1 | Cognitive task analysis.mp. |  |
|  | 2 | Cognitive training.mp. |  |
|  | 3 | (Cognitive adj2 simulation).mp. |  |
|  | 4 | Cognitive skills.mp. |  |
|  | 5 | 1 or 2 or 3 or 4 |  |
|  | 6 | Surg*.mp. |  |
|  | 7 | Operat*.mp. |  |
|  | 8 | 6 or 7 |  |
|  | 9 | 5 and 8 |  |
|  | | | |
| **EMBASE** | 1 | Cognitive task analysis.mp. |  |
|  | 2 | Cognitive training.mp. |  |
|  | 3 | (Cognitive adj2 simulation).mp. |  |
|  | 4 | Cognitive skills.mp. |  |
|  | 5 | 1 or 2 or 3 or 4 |  |
|  | 6 | Surg*.mp. |  |
|  | 7 | Operat*.mp. |  |
|  | 8 | 6 or 7 |  |
|  | 9 | 5 and 8 |  |
|  | | | |
| **Web of Science** | 1 | TS=(“Cognitive task analysis”) |  |
|  | 2 | TS=(“Cognitive training”) |  |
|  | 3 | TS=(Cognitive NEAR/2 simulation) |  |
|  | 4 | TS=(“Cognitive skills”) |  |
|  | 5 | #1 OR #2 OR #3 OR #4 |  |
|  | 6 | TS=(Surg*) |  |
|  | 7 | TS=(Operat*) |  |
|  | 8 | #6 OR #7 |  |
|  | 9 | #5 AND #8 |  |
|  | | | |
| **CENTRAL** | 1 | “Cognitive task analysis” |  |
|  | 2 | “Cognitive training” |  |
|  | 3 | Cognitive NEAR/2 simulation |  |
|  | 4 | “Cognitive skills” |  |
|  | 5 | #1 OR #2 OR #3 OR #4 |  |
|  | 6 | Surg* |  |
|  | 7 | Operat* |  |
|  | 8 | #6 OR #7 |  |
|  | 9 | #5 AND #8 |  |

| **Supplementary Table 2.** Search strategy for grey literature | |
| --- | --- |
| **Grey Search** | **Findings** |
| ProQuest | 4 dissertation theses^25-28^ |
| Open Grey | 0 |
| Google - .docx cognitive task analysis surgery | 0 |
| Google - .pdf cognitive task analysis surgery | 0 |
| Clinicaltrials.org | 0 |
