# Supplementary material for: Cognitive task analysis-based training in surgery: a meta-analysis
Source: BJS Open. 2021 Dec 14;5(6):zrab122. doi: 10.1093/bjsopen/zrab122 (PMC8669793; doi:10.1093/bjsopen/zrab122)
Supplement: zrab122_Supplementary_Data [file zrab122_supplementary_data.zip › Supplementary_Tables_3-5.docx]

| **Supplementary Table 3.** Study design of included articles | | | | | | |
| --- | --- | --- | --- | --- | --- | --- |
| **Reference (publication year)** | **Study type** | **Study participants (intervention, control)** | **Surgical procedure** | **CTA methodology** | **CTA-based training intervention(s)** | **Control** |
| Bathalon et al. (2005)^13^ | Randomised controlled trial | 44 medical students (13,16) + 15 in mental imagery and kinesiology | Open cricothyrotomy | Not reported | CTA-based course using kinesiology | Traditional advanced trauma life support (ATLS) course |
| Bhattacharyya et al. (2017)^14^ | Randomised controlled trial | 16 surgical trainees (8,8) | Diagnostic knee arthroscopy | Modified Delphi technique with three expert knee surgeons | Downloadable multimedia learning tool (+ flowchart of steps) | Flowchart of steps only |
| Bhattacharyya et al. (2018)^15^ | Randomised controlled trial | 22 medical students (11,11) | Intramedullary femoral nailing | Modified Delphi technique with three expert trauma and orthopaedic surgeons | Web-based multimedia tool | Traditional operative technical manual |
| Campbell et al. (2011)^16^ | Randomised controlled trial | 26 medical students and surgical trainees  (12,14) | Open cricothyrotomy | Structured CTA interviews with six expert trauma surgeons | CTA-based curriculum (same as traditional but using CTA-based content) | Traditional curriculum (including didactic lecture, observation of a demonstration and guided practice of procedure) |
| Kowalewski et al. (2017)^17^ | Randomised controlled trial | 46 medical students (23,23) | Laparoscopic cholecystectomy | Not reported | CTA-based simulated mobile phone application (Touch Surgery) + procedural task modules | Procedural task modules on virtual reality surgical simulation system only |
| Logishetty et al. (2020)^18^ | Randomised controlled trial | 36 surgical trainees (18,18) | Anterior approach total hip arthroplasty | Modified Delphi technique with four expert surgeons | Web-based multimedia tool | Traditional operative technical manual and video of the procedure |
| Luker at al. (2008)^19^ | Observational study | 9 surgical trainees | Flexor tendon repair | Series of three interviews with three expert surgeons | Multimedia computer programme | Traditional learning (intervention group served as their own control) |
| Shariff et al. (2014)^20^ | Randomised controlled trial* | 43 surgical trainees  (25,18) | Open and laparoscopic anterior resection | Not reported | Web-based multimedia tool | CTA-based study day (including interactive lectures) |
| *(continued on next page)* | | | | | | |

| **Supplementary Table 3** (*continued*) | | | | | | |
| --- | --- | --- | --- | --- | --- | --- |
| **Reference (publication year)** | **Study type** | **Study participants (intervention, control)** | **Surgical procedure** | **CTA methodology** | **CTA-based training intervention(s)** | **Control** |
| Sugand et al. (2016)^21^ | Observational study | 27 medical students | Intramedullary femoral nailing | Not Reported | CTA-based simulated mobile phone application (Touch Surgery) | No control |
| Sullivan et al. (2007)^22^ | Randomised controlled trial | 20 surgical trainees  (9,11) | Percutaneous tracheostomy | 1) Three percutaneous tracheostomy experts were interview by trained CTA analyst | CTA-based curriculum | Traditional curriculum (including didactic lectures and demonstration on model) |
|  |  |  |  | 2) Probed on cognitive decisions by using pre-recorded video of the surgeon performing a PT |  |  |
| Velmahos et al. (2004)^23^ | Randomised controlled trial | 26 surgical trainees (12,14) | Central venous catheterisation | Interviews with two CVC experts by two expert CTA analysts | CTA-based course | Traditional clinical teaching |
| Vestermark et al. (2019)^24^ | Randomised controlled trial | 12 surgical trainees (6,6) | Robotic arm-assisted unicompartmental knee arthroplasty | Not reported | CTA-based simulated mobile phone application (Touch Surgery) | Traditional paper-based surgical reference guide |
| PT, percutaneous tracheostomy; CVC, central venous catheterisation.  *Participants were randomised to different CTA modes of delivery, not CTA versus a non-CTA control. | | | | | | |

| **Supplementary Table 4.** Assessment and summary of results of included articles | | | | |
| --- | --- | --- | --- | --- |
| **Reference (publication year)** | **Assessment method** | **Outcome measurements** | **Summary of results**  (Mean ± S.D) | **MERSQI score** |
| Bathalon et al. (2005)^13^ | Performance of open cricothyrotomy on model | Technical performance score using 25-point OSCE | No overall significant difference found between ATLS control group and CTA-based kinesiology group (CTA: 19.3 ± 2.9 vs ATLS: 18.2 ± 2.5) | 12.5 |
| Bhattacharyya et al. (2017)^14^ | Performance on high-fidelity, phantom knee arthroscopy simulator | Validated Arthroscopic Surgical Skill Evaluation Tool (ASSET) score  (measures technical performance of diagnostic knee arthroscopy) | The CTA-based learning tool group achieved greater mean ASSET scores compared to the control group (CTA: 19.5 ± 3.7 vs Con: 10.6 ± 2.32, p=0.002) | 15.5 |
| Bhattacharyya et al. (2018)^15^ | Validated Touch Surgery femoral intramedullary nailing module via assessment mode (SBA questions) | Assessment test score  (measures knowledge and understanding of procedure) | The CTA-based multimedia group and control group both significantly (p<0.05) increased their scores in 4/4 and 3/4 touch surgery FIN assessment modules respectively. CTA-based multimedia scored 10-21% higher in each module assessment compared to the control group (p<0.001) | 14.5 |
| Campbell et al. (2011)^16^ | 1) Procedural cognitive knowledge test  2) Performance of open cricothyrotomy on model | 1) Procedural cognitive knowledge test scores (measures understanding of both actions and decisions needed to successfully perform procedure) 2) Technical and procedural performance of open cricothyrotomy using procedural checklist (measures performance of the action and decision steps to be performed successfully in the correct order) | The CTA-based curriculum post-training test score showed no significant difference to the traditional based curriculum (CTA: 18.17 ± 3.61 vs Con: 16.71 ± 2.64, p=0.59). Although, the CTA-based curriculum outperformed the control group in technical and procedural performance of open cricothyrotomy (CTA: 17.75 ± 2.34 vs Con: 15.14 ± 2.48, p=0.006) | 12.5 |
| Kowalewski et al. (2017)^17^ | Lap Mentor II laparoscopic virtual reality simulator | Technical performance of laparoscopic cholecystectomy using Lap Mentor II in-simulator metrics | No significant difference was found in the group who received the CTA-based simulation application in addition to the Lap Mentor II training for both final operation time (CTA: 757.5 ± 307.9 s vs Con: 662.6 ± 259.4 s, p=0.265) and times attempted to complete the procedure without any serious complications (CTA: 1.9 ± 2.7 vs Con: 2.4 ± 1.9, p=0.462) | 14.5 |
| *(continued on next page)* | | | | |

| **Supplementary Table 4** (*continued)* | | | | |
| --- | --- | --- | --- | --- |
| **Reference (publication year)** | **Assessment method** | **Outcome measurements** | **Summary of results**  (Mean ± S.D) | **MERSQI score** |
| Logishetty et al. (2020)^18^ | 1) Procedural knowledge test (10 MCQ questions) 2) Performance of acetabular cup orientation on model  3) Performance of the AA-THA procedure using fully immersive virtual reality system | 1) Procedural knowledge test score  2) Cup orientation by measuring mean combined error from target of 40° inclination and 20° anteversion 3) Technical performance of AA-THA using in-simulation metrics and investigator; incorrect instrument selected, or incorrect step taken, time taken to complete procedure, and number of times assistance required from investigator | The CTA-based group attained higher MCQ test results (CTA: 6 ± 1 vs Con: 4 ± 1, p<0.005) and orientated the acetabular cup placement more accurately than the traditional control group (mean combined error, CTA: 16° vs Con: 24°, p<0.005). The CTA-based tool group achieved better in-simulation AA-THA procedure metrics including; 10 minutes quicker to complete procedure (CTA: 28 ± 9 vs Con: 38 ± 8, p<0.005), 69% fewer instrument selection error (CTA: 29 ± 8 vs Con: 49 ± 10) and 92% fewer prompts for the following step (CTA: 13 ± 4 vs Con: 25 ± 7) compared to the traditional group. | 15.5 |
| Luker at al. (2008)^19^ | Talk-aloud protocol | Total knowledge and cognitive decision points of procedure score (including advantages and disadvantages of procedure) | The CTA-based multimedia group statistically significantly improved scores by 34 in total knowledge and by 19.4 in elicitation of the advantage and disadvantages of the procedure (p<0.01). The control group did not significantly improve following traditional learning (13.2 and 8.4 respectively) | 11 |
| Shariff et al. (2014)^20^ | 40-point procedural cognitive knowledge test  (30 questions randomly provided from a bank of 200 questions – 20 MCQ and 10 short-answer) | Procedural cognitive knowledge test score | The CTA-based multimedia group significantly increased their mean score after CTA-based training (pre-test: 20.95 ± 5.84 vs post-test: 27.55 ± 6.36, p<0.01). The CTA-based study day group also improved their mean score after the intervention (pre-test: 20.52 ± 4.93 vs post-test: 25.41 ± 5.05, p<0.01) | 14 |
| Sugand et al. (2016)^21^ | Procedural knowledge test (MCQ) | Procedural knowledge test score | The CTA-based simulation app showed a significant training effect with participants achieving an overall mean 83-94% in assessed modules post-training compared to their pre-training scores (p<0.001). After the sixth attempt students achieved MCQ results non-statistically different compared to expert surgeons (Nov: 90-98% vs Exp: 89-95%, no p value provided) | 13 |
| *(continued on next page)* | | | | |

| **Supplementary Table 4** (*continued)* | | | | |
| --- | --- | --- | --- | --- |
| **Reference (publication year)** | **Assessment method** | **Outcome measurements** | **Summary of results**  (Mean± S.D) | **MERSQI score** |
| Sullivan et al. (2007)^22^ | 1) Think-out-loud protocol  2) Performance of percutaneous tracheostomy on model; 1 month and 6 months post-intervention | 1) Procedural cognitive knowledge score using 40-point assessment tool  2) Technical performance of percutaneous tracheostomy score using 50-point checklist | The CTA-based curriculum group achieved higher scores in describing the cognitive procedural points of PT (CTA: 25.4 ± 5.3 vs Con: 19.2 ± 2.0, p=0.004). The CTA-based group also outperformed the traditional curriculum group on technical performance of the PT at 1 month (CTA: 43.5 ± 3.7 vs Con: 35.2 ± 3.9, p=0.001) and 6 months (CTA: 39.4 ± 4.2 vs Con: 31.8 ± 5.8, p=0.004) | 12.5 |
| Velmahos et al. (2004)^23^ | 1) 15 question procedural knowledge test (MCQ) 2) Performance of CVC on real patient | 1) Procedural knowledge test score 2) Technical performance of CVC score using 14-point step-by-step checklist and complications when performing procedure on patient | The CTA-based course group acquired significantly greater procedural knowledge post-test scores compared to the traditional group (CTA: 11.0 ± 1.86 vs Con: 8.64 ± 1.82, p=0.03) and significantly performed the CVC procedure to a higher standard score when compared to the traditional group (CTA: 12.6 ± 1.1 vs Con: 7.5 ± 2.2, p<0.001). All CVC complications were recorded in the traditional group (sample size too small to make conclusions) | 14 |
| Vestermark et al. (2019)^24^ | 25 question procedural knowledge test (MCQ); immediately post-training and 3-weeks post-training | Procedural knowledge test score | The CTA-based simulation group significantly increased their MCQ scores post-training by 22% (p=0.001), whereas the control paper-based learning group increased their knowledge by only 10% (p=0.09). Recall (3 weeks post-training) was better in the CTA-based simulation group when compared to the control group | 13.5 |
| Mean ± S.D = mean average ± standard deviation.  Con, control; FIN, femoral intramedullary nailing; SBA, single best answer; OSCE, objective structured clinical examination; ASSET, arthroscopic surgical skill evaluation tool; ATLS, advanced trauma life support; MCQ, multiple choice questions; AA-THA, anterior approach total hip arthroplasty; NOV, novice; EXP, expert; PT, percutaneous tracheostomy; CVC, central venous catheterisation. | | | | |

| **Supplementary Table 5.** Summary of included articles MERSQI scores | | | | | | | | | | | | | | |  |
| --- | --- | --- | --- | --- | --- | --- | --- | --- | --- | --- | --- | --- | --- | --- | --- |
|  |  | **Articles** | | | | | | | | | | | |  |  |
| **Domain** | **Item (maximum points)** | Bathalon et al.^13^ | Bhattacharyya et al. (2017)^14^ | Bhattacharyya et al. (2018)^15^ | Campbell et al.^16^ | Kowalewski et al.^17^ | Logishetty et al.^18^ | Luker et al.^19^ | Shariff et al.^20^ | Sugand et al.^21^ | Sullivan et al.^22^ | Velmahos et al.^23^ | Vestermark et al.^24^ | | |
| Study design | Study design (3) | 3 | 3 | 3 | 3 | 3 | 3 | 1.5 | 3 | 1.5 | 3 | 3 | 3 | | |
| Sampling | Number of institutions studied (1.5) | 0.5 | 0.5 | 0.5 | 0.5 | 0.5 | 1.5 | 0.5 | 0.5 | 0.5 | 0.5 | 0.5 | 0.5 | | |
|  | Response rate (1.5) | 1.5 | 1.5 | 1.5 | 1.5 | 1.5 | 1.5 | 1.5 | 1 | 1.5 | 1.5 | 1.5 | 1.5 | | |
| Type of data | Type of data (3) | 3 | 3 | 3 | 3 | 3 | 3 | 3 | 3 | 3 | 3 | 3 | 3 | | |
| Validity of evaluation instrument | Internal structure (1) | 0 | 1 | 0 | 0 | 0 | 0 | 0 | 1 | 0 | 0 | 0 | 0 | | |
|  | Content (1) | 0 | 1 | 1 | 0 | 1 | 1 | 0 | 0 | 1 | 0 | 0 | 1 | | |
|  | Relationships to other variables (1) | 0 | 1 | 1 | 0 | 1 | 1 | 0 | 1 | 1 | 0 | 0 | 0 | | |
| Data analysis | Appropriateness of analysis (1) | 1 | 1 | 1 | 1 | 1 | 1 | 1 | 1 | 1 | 1 | 1 | 1 | | |
|  | Complexity of analysis (2) | 2 | 2 | 2 | 2 | 2 | 2 | 2 | 2 | 2 | 2 | 2 | 2 | | |
| Outcomes | Outcomes (3) | 1.5 | 1.5 | 1.5 | 1.5 | 1.5 | 1.5 | 1.5 | 1.5 | 1.5 | 1.5 | 3 | 1.5 | | |
| **Total score** | **(18)** | 12.5 | 15.5 | 14.5 | 12.5 | 14.5 | 15.5 | 11 | 14 | 13 | 12.5 | 14 | 13.5 | | |
